# Development of Suberinic Acids-Bonded Medium-Density Particleboard

**DOI:** 10.3390/polym18040487

**Published:** 2026-02-14

**Authors:** Ramunas Tupciauskas, Andris Berzins, Gunars Pavlovics, Rudolfs Berzins, Martins Andzs, Janis Rizikovs

**Affiliations:** 1 Laboratory of Biorefinery, Latvian State Institute of Wood Chemistry, Dzerbenes 27, 1006 Riga, Latvia; andris.berzins@kki.lv (A.B.); pavlovichs@inbox.lv (G.P.); rudolfs.berzins@kki.lv (R.B.); martins.andzs@kki.lv (M.A.); janis.rizikovs@kki.lv (J.R.); 2 Faculty of Forest and Environmental Sciences, Latvia University of Life Sciences and Technologies, Akademijas 11, 3001 Jelgava, Latvia

**Keywords:** wood sawdust, recycled particleboard, bio-based adhesive, suberinic acids, green composites, particleboard, properties

## Abstract

This study focuses on the development of wood-based particleboard that address resource efficiency, environmental sustainability, and health-related concerns. The conventional particleboard industry relies on synthetic, predominantly formaldehyde-based adhesives, which pose environmental, health, and end-use risks. Rising raw material prices, regulatory restrictions, and increasing competition in the wood-processing sector have further highlighted the importance of alternative biomass resources for particleboard production. In response to these challenges, this study investigates the suitability of available sawdust resources derived from the production residues of cellular wood materials and recycled particleboards, combined with natural suberinic acids mixture obtained from birch outer bark as a binder. The effects of furnish structure, binder content (15–21%), pressing temperature (190–220 °C), pressing rate (0.9–1.7 min/mm), and board density (650–850 kg/m^3^) on the resulting particleboard properties were evaluated. The results demonstrate that it is possible to meet the requirement values for thickness swelling (≤17%) and internal bonding strength (≥0.40 N/mm^2^) specified for interior fitment boards, including furniture applications according to EN 312, Type P2. The bending properties of the best-performing particleboards are very close to the requirement values (MOE ≥ 1800 N/mm^2^, MOR ≥ 11 N/mm^2^), indicating the potential for further improvement at the target density range. Furnish structure, board thickness, density, and pressing temperature were identified as the most influential factors affecting the final board properties.

## 1. Introduction

The conventional global production of wood-based particleboards has shown a steady increase, exceeding 100 million m^3^ per year since 2021 [1]. Particleboard is an engineered material widely used in furniture, construction, and packaging composed of wood or other lignocellulosic particles bonded with synthetic adhesives that typically contain carcinogenic formaldehyde. This raises significant health concerns, especially in indoor environments where air-quality standards must be met [2]. Consequently, the development of eco-friendly adhesives—preferably derived from natural resources—has become increasingly important. In the field of natural biomass-based adhesive development, numerous attempts have been made to incorporate starch [3], proteins (soy, gluten, casein) [4], lignins (alkaline, hydrolysis, organosolv, lignosulfonate, etc.) [5], tannins [6], and various organic acids [7] into particleboard production while achieving the required performance properties. Studies on particleboards bonded with alternative bio-based adhesives (including on soy protein, lignin, tannin, and starch) report physical and mechanical properties comparable to those of boards produced with conventional urea–formaldehyde resins [8]. There is also considerable interest in developing binder-less particleboards that require no additional adhesives [9]. However, this approach is generally feasible only for high-density boards (>1000 kg/m^3^) [10]. Efforts to reduce environmental impact by improving redesigning synthetic adhesives for particleboard have likewise been explored [11,12].

Suberinic acids (SA) obtained from the outer bark of birch (*Betula*) have been investigated at the Latvian State Institute of Wood Chemistry [13], demonstrating good adhesive properties and potential suitability for particleboard production using alder [14] and birch [15,16] wood particles. A detailed structural analysis of SA has revealed the presence of monosaccharides, polyphenolics, and—mostly importantly—a high content of saturated long-chain fatty acids containing epoxy and hydroxyl groups, which contribute to their favorable adhesive performance in wood-composite bonding via esterification [17]. However, the compatibility of SA with other raw materials has not yet been investigated, particularly at lower panel densities. To date, only one study has reported an attempt to use residues from ethanol–alkaline SA isolation as a binder mixed with different types of particles from *Pinus sylvestris* L. for particleboard production [18].

Another challenge for the particleboard industry is the availability of raw materials, driven by competition among wood-product sectors, supply limitations, and rising costs. One key solution is the reuse of wood after the end of life of various wood-based products, which is increasingly approaching industrial relevance. It has been reported that particleboard and the core layer of oriented strand board can be fully substituted with recycled wood particles while maintaining the required property values according to relevant standards [19]. Another viable approach to fulfill the raw material demand is the use of available agro–industrial residues such as straw, stalks, bagasse, grasses, etc. Numerous studies have demonstrated the feasibility of manufacturing particleboards from agricultural residues, often achieving property values comparable to those of conventional particleboards [20]. These approaches support the sustainable development of particleboard production by promoting economic growth, social inclusion, and environmental protection.

The performance of particleboards produced from annual plant by-products bonded with casein adhesive has also demonstrated promising potential for applications such as furniture or door panels [21]. In addition to these alternatives, large quantities of sawmill by-products—particularly sawdust—are available and hold significant potential for particleboard manufacturing [22]. Several decades ago, a cellular wood material (CWM) was developed as a lightweight wood product for furniture, door components, and transportation applications [23]. However, due to the sawn longitudinal grooves required in its production, more than 40% of the processed logs are converted into sawdust, creating a substantial by-product stream suitable for further utilization.

Taking into account the issues outlined above, this study investigates particleboards produced using different types of sawdust and SA-based binder derived from birch outer bark. For the first time, medium-density particleboards were manufactured using a combination of sawdust generated during CWM production from pine wood (*Pinus sylvestris*), recycled particles from used particleboards, and a SA binder. The production of SA binder and the experimental design were based on previous research involving particleboard made of conventional birch wood particles [15]. The resulting particleboards were evaluated for their potential suitability for interior fitments (including furniture) intended for dry condition use in accordance with European standard requirements EN 312, Type P2 [24]. The findings provide a data-driven assessment of the suitability of the selected sawdust types and SA binder for particleboard production and highlight key directions for further optimization of their mechanical performance.

## 2. Materials and Methods

### 2.1. Raw Materials

**Figure 1:**
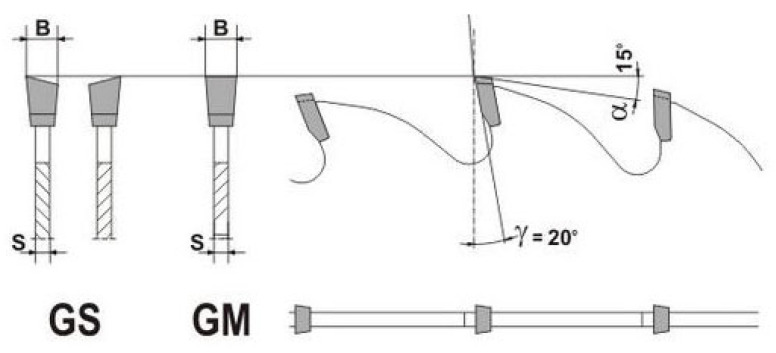
Tooth angles of circular saws GS (LS-/) and GM (LS-|).

Three different furnishes of wood particles were used in this study for board production and evaluation. Two of the furnishes were prepared by a longitudinal inner sawing of dried pine (*Pinus sylvestris*) boards (20 mm × 100 mm × 1000 mm) using two different circular sawblades. One sawblade had 24 teeth (Z24) with a 20° top-bevel angle (Figure 1, GS), while the other had 18 teeth (Z18) with a flat-top configuration (0°) (Figure 1, GM). Both sawblades had the same diameter (250 mm) and tooth width (3.2 mm). In the following text, the sawdust produced using the GS sawblade is referred to as LS-/ and the sawdust produced by GM sawblade as LS-|.

The third particle furnish was supplied by the local particleboard manufacturer Kronospan Latvia and consisted of recycled particleboard particles in two fractions. Fine particles (<2 mm) and coarse particles (55% > 2 mm) were received without any added binder. However, since these particles originated from recycled particleboards, it is assumed that they contain residual cured adhesive and additives. As the delivered industrial furnish consisted solely of recycled particles, it is hereafter referred to as RE. The dimensional distribution of the furnish particles is presented in Table 1.

### 2.2. Binder Preparation

Ethanol-extracted birch outer bark was ground to pass a 2 mm sieve and hydrolytically depolymerized in a 4% KOH solution for 30 min, as described in Ref. [25]. The filtered wet product with a moisture content (MC) of approximately 75% was used as the SA-based binder (solubility in tetrahydrofuran ~54%, monosaccharides 11.4%, ω-hydroxy acids 17.1%, α,ω-diacids—20.8%, triterpenes 11.0%, acid number ~49 mg KOH/g) without any additional additives.

### 2.3. Production of Particleboards

Three-layer particleboards were produced using the three furnishes described above. To utilize all fractions of sawn LS particles, the face layers were formed from particles with a fraction < 1 mm, while the core layer consisted of particles >1 mm. Based on a previous study [15], 21–25 wt.% (dry basis) of wet SA binder was added to the LS furnishes, and 15–21 wt.% was added to the RE furnish. The blended furnishes were then dried (MC < 2%) and hot-pressed at varying temperatures (190–220 °C) and pressing times (0.9–1.7 min/mm) to obtain particleboards with final dimensions of 9 mm × 300 mm × 300 mm, with target densities ranging from 650 to 850 kg/m^3^. The pressing cycle for all board types consisted of four stages:

1. Pressing at maximum pressure (3 MPa) to reach the target thickness for 3.5 min;
2. Releasing the pressure at 0.2 MPa for 30 s;
3. Pressing at reduced pressure (<2 MPa) to stabilize the thickness for 3 min;
4. Pressing under minimum pressure (0.5 MPa) until the end of the set pressing time.

**Figure 2:**
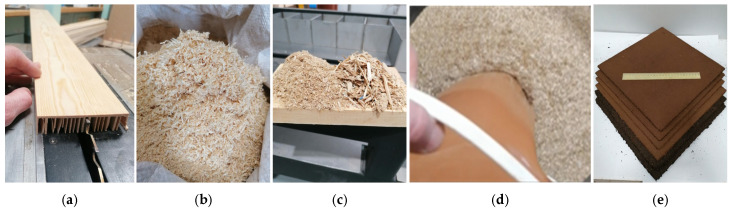
Flow diagram representing preparation of LS sawdust (**a**), shape of LS particles (**b**), shape of RE particles (**c**), addition of binder (**d**), and obtained particleboards (**e**).

One particleboard sample was produced for each furnish type, and the pressing conditions are summarized in Table 2. Distance bars were used during the hot-pressing for consistent board thickness. The particleboard production workflow is shown in Figure 2.

The produced particleboard samples were conditioned in a climate chamber under controlled conditions (temperature 20 ± 2 °C and relative humidity 60 ± 5%) until equilibrium moisture content.

### 2.4. Evaluation of Particleboards

The obtained particleboards were characterized according to the relevant standards by determining the density [26], modulus of elasticity (MOE), and modulus of rupture (MOR) in a three-point bending test [27]; tensile strength perpendicular to the board surface (IB) [28]; thickness swelling and water absorption (TS/WA) after 24 h immersion in water [29]; and face withdrawal of screw resistance (WSR) [30]. In addition, a surface optical morphology was performed with stereoscopic light microscopy (Leica Microsystems Leica S9, Wetzlar, Germany) equipped with the image acquisition software LAS Core V 4.12.0 analyzing face and cross sections of the boards. Observations were carried out in reflected LED light mode at 2× magnification. Mechanical tests (MOE, MOR, IB, FWS) were performed using a ZWICK/Roell Z010 (Ulm, Germany) universal machine.

Specimen dimensions were 200 mm × 50 mm for bending tests and 50 mm × 50 mm for all other property evaluations. Five specimens were tested for each property to calculate the average value for each particleboard type. The resulting board properties were compared with the European requirements specified in EN 312 for particleboards intended for interior fitments (including furniture) for use in dry conditions (Type P2) [24]. The influence on the selected factors on the mean property values was analyzed at a confidence level α = 0.05 by using one-way ANOVA and CORRELATION tools in Microsoft Excel.

## 3. Results

### 3.1. Properties of Obtained Particleboards

The obtained particleboards exhibited a brown coloration due to the SA binder and showed good machinability and surface roughness (Figure 2e). An initial assessment of LS particle fractions and pressing time indicated that particles >2 mm yielded the highest bending properties (MOE 900 N/mm^2^, MOR 6 N/mm^2^). In contrast, pressing times of 0.8 and 1.2 min/mm did not produce significant differences in the evaluated properties. Based on these findings and the fractional composition of the LS furnish (Table 1), subsequent boards were manufactured as three-layer structures: the core layer was composed of particles >1 mm, and the face layers were composed of particles < 1 mm. In addition to varying pressing conditions, four sample groups were made from both LS furnishes with the <0.5 mm fraction removed. The results of all measured particleboard properties as a function of the tested variables are summarized in Table 3.

The density values of the obtained particleboards ranged from 620 to 960 kg/m^3^ (Table 3), exceeding the initially targeted density range. This variation occurred because different particle furnishes—with distinct particle shapes and size distributions (Table 1)—were used. During experimental particleboard preparation, the furnish mass required to achieve the target density was adjusted individually for each furnish type, which subsequently resulted in the observed density values.

#### 3.1.1. Resistance to Water

After 24 h immersion in water, the WA values of the obtained particleboards ranged from 49% to 109% (Table 3). The variation in WA values was substantial and was primarily dependent on the board density (r = −0.73). A moderate influence of pressing temperature was also observed (r = −0.39), with lower WA values recorded for boards hot-pressed at a higher temperature (220 °C). On average, boards made from LS particles exhibited lower WA (76%) than those made from RE particles (87%), indicating the influence of particle geometry. However, this effect was also linked to binder content. For example, when comparing boards made from different furnishes but with the same binder content, WA values did not differ significantly (samples 7, 15, and 27; Table 3).

**Figure 3:**
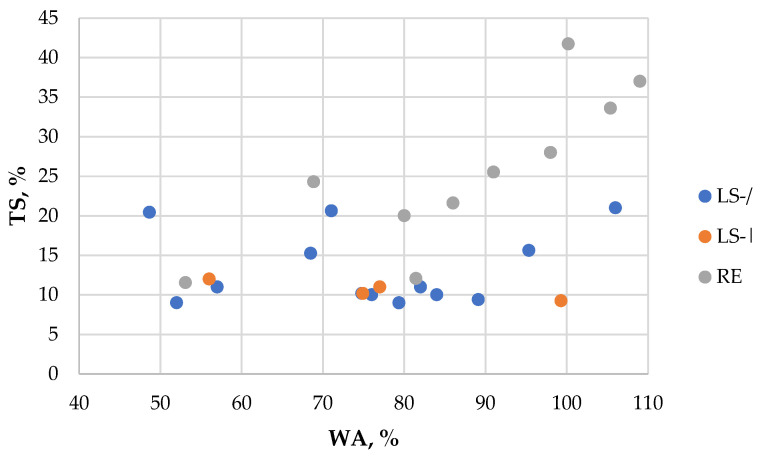
TS vs. WA of the obtained particleboards depending on furnish type.

No significant differences in WA were observed between the LS-/ and LS-| furnishes (samples 6 vs. 14 and 7 vs. 15; Table 3). A similar trend was found when the fine fraction was removed from both LS furnishes (samples 7 vs. 8 and 15 vs. 16; Table 3). A strong positive correlation (r = 0.52) between WA and TS values across the different furnishes is shown in Figure 3. In addition, strong negative correlations were identified between WA and MOR (r = −0.70) and between WA and IB (r = −0.71), indicating that higher water absorption is associated with lower mechanical performance.

**Table 3:** Properties of obtained particleboards bonded by SA-based adhesive.

No	Sample ^1^	Density (kg/m^3^)	WA (%)	TS (%)	MOE (N/mm^2^)	MOR (N/mm^2^)	IB (N/mm^2^)	WSR (N)
1	LS-/-21-1.0-210	665 (54) ^2^	95 (15)	15.6 (1.1)	445 (110)	2.1 (0.3)	0.16 (0.04)	n.d. ^3^
2	LS-/-21-1.0-220	670 (39)	79 (5)	9.0 (2.5)	600 (45)	2.9 (0.2)	0.25 (0.06)	n.d.
3	LS-/-21-1.0-210	775 (44)	68 (8)	15.3 (1.8)	955 (175)	4.5 (0.5)	0.27 (0.07)	n.d.
4	LS-/-21-1.4-200	820 (39)	71 (7)	20.6 (0.9)	1155 (190)	6.6 (1.2)	0.73 (0.27)	n.d.
5	LS-/-21-1.4-200	960 (35)	49 (7)	20.4 (1.2)	1740 (150)	10.3 (1.2)	0.94 (0.13)	n.d.
6	LS-/-21-1.5-220	650 (32)	89 (6)	9.4 (1.0)	390 (90)	2.0 (0.5)	0.41 (0.11)	251 (70)
7	LS-/-21-1.5-220	715 (39)	75 (5)	10.2 (1.0)	870 (120)	4.7 (0.7)	0.63 (0.10)	372 (59)
8	LS-/>05-21-1.5-220	700 (46)	76 (11)	10.0 (0.5)	720 (195)	4.5 (0.9)	0.79 (0.24)	313 (72)
9	LS-/>05-21-1.5-220	805 (34)	57 (8)	11.0 (0.8)	1095 (170)	5.9 (0.9)	1.19 (0.17)	481 (27)
10	LS-/-21-1.5-220	748 (44)	52 (9)	9.2 (0.5)	1120 (250)	7.9 (1.6)	1.54 (0.31)	505 (150)
11	LS-/-21-1.7-220	634 (45)	84 (14)	9.8 (0.2)	550 (90)	4.1 (0.7)	0.74 (0.10)	321 (90)
12	LS-/-25-1.5-220	660 (33)	82 (10)	11.0 (0.5)	635 (130)	4.8 (1.0)	0.85 (0.23)	303 (75)
13	LS-/-25-4.5-190	649 (41)	106 (11)	21.2 (3.1)	365 (75)	3.2 (0.8)	0.56 (0.16)	424 (70)
14	LS-|-21-1.5-220	625 (47)	99 (12)	9.2 (1.3)	440 (90)	2.7 (0.8)	0.29 (0.09)	209 (93)
15	LS-|-21-1.5-220	730 (51)	75 (11)	10.2 (1.0)	740 (165)	4.4 (0.7)	0.66 (0.12)	327 (93)
16	LS-|>05-21-1.5-220	700 (66)	77 (14)	11.0 (0.0)	700 (220)	3.9 (0.8)	0.46 (0.17)	243 (93)
17	LS|>05-21-1.5-220	815 (53)	56 (11)	12.0 (0.7)	1245 (340)	7.4 (1.8)	0.80 (0.16)	390 (115)
18	RE-15-0.9-200	730 (23)	100 (4)	41.7 (2.5)	1452 (76)	2.9 (0.2)	0.32 (0.09)	n.d.
19	RE-15-0.9-200	655 (15)	91 (3)	25.5 (1.0)	1483 (346)	6.2 (1.4)	0.37 (0.21)	n.d.
20	RE-15-0.9-220	660 (62)	86 (13)	21.6 (1.0)	1365 (124)	5.2 (1.4)	0.48 (0.02)	n.d.
21	RE-15-0.9-220	750 (32)	69 (4)	24.3 (3.2)	2040 (94)	10.1 (0.7)	0.72 (0.15)	n.d.
22	RE-15-1.0-200	650 (27)	105 (8)	33.6 (1.7)	1153 (72)	2.3 (0.4)	0.26 (0.06)	n.d.
23	RE-15-1.5-200	665 (18)	109 (12)	37.0 (5.3)	1160 (140)	5.3 (0.6)	0.46 (0.17)	511 (160)
24	RE-15-1.1-220	644 (19)	80 (11)	20.1 (4.1)	1170 (75)	5.9 (0.5)	0.62 (0.11)	545 (95)
25	RE-15-1.5-220	653 (18)	98 (21)	28.4 (8.7)	1185 (190)	6.1 (0.7)	0.62 (0.19)	513 (150)
26	RE-21-1.5-220	620 (38)	81 (11)	12.1 (0.7)	819 (118)	3.6 (0.3)	0.57 (0.08)	384 (81)
27	RE-21-1.5-220	720 (25)	53 (7)	11.5 (0.8)	1639 (67)	8.4 (0.5)	0.94 (0.16)	588 (60)
Standard requirement (EN 312 Type P2)			–	≤17 ^4^	≥1800	≥11	≥0.40	–

^1^ The sample code includes the following values: furnish particle details (Section 2.1), SA binder content (%), pressing rate (min/mm), pressing temperature (°C); ^2^ values in parenthesis are standard deviation; ^3^ n.d. not detected; ^4^ Type P3. The specified requirement values of Types P2 and P3 apply to boards with a thickness in the range of >6–13 mm.

The measured TS values of the obtained particleboards varied substantially, ranging from 9% to 42% (Table 3). A strong negative correlation was observed between TS and pressing temperature (r = −0.67), indicating that higher pressing temperatures improved TS performance. On average, boards made from LS particles exhibited approximately two-fold lower TS compared with boards from RE particles, which, as in the case of WA, can be attributed to the different binder contents. However, similar to the WA results, when comparing boards manufactured from the three different furnish particle types under identical conditions and density (samples 7, 15, and 27; Table 3), the difference in TS values was insignificant. Likewise, removing the fine fraction from LS particles resulted in an insignificant difference on TS values (samples 7 vs. 8 and 15 vs. 16; Table 3).

Increasing the binder content from 21% to 25% in boards made from LS furnish resulted in only insignificant differences in both WA and TS values (samples 6 vs. 12; Table 3). However, in the case of RE furnish, raising the binder content from 15% to 21% improved water resistance, with the effect being statistically significant in the case of TS (samples 25 vs. 26; Table 3).

#### 3.1.2. Resistance to Bending

**Figure 4:**
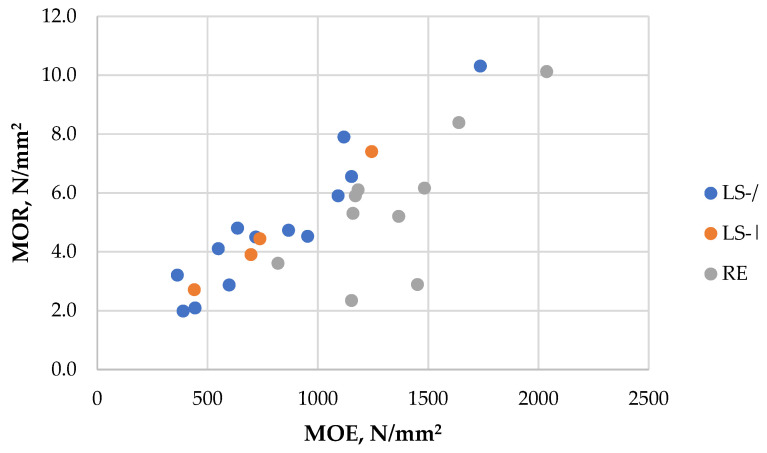
MOE vs. MOR of the obtained particleboards depending on furnish.

The MOR values of the produced particleboards ranged from 2.0 N/mm^2^ to 10.3 N/mm^2^ (Table 3), showing a strong dependence on board density (r = 0.65). The MOE values varied between 360 N/mm^2^ and 2040 N/mm^2^ (Table 3) and, although the dependence on density was lower, the correlation remained strong (r = 0.51). A very strong correlation (r = 0.79) obtained between MOR and MOE of the boards (Figure 4) indicates that the boards exhibit high stiffness but relatively low strength. A slight negative correlation was observed between MOE and pressing rate (r = −0.40). Some variation in MOR and MOE was observed depending on pressing conditions and LS furnish type (including the presence or absence of fine fractions). In several cases, the differences were statistically significant, although the absolute changes in bending properties remained modest. On average, boards manufactured from RE particles achieved higher MOE and MOR values than those made from LS particles (e.g., samples 7 and 15 vs. 27; Table 3). It is also noteworthy that the highest bending values for LS-based boards were obtained at a high density (sample 5; Table 3), whereas the RE-based board with the highest bending performance (sample 21; Table 3) reached these values at a density of 750 kg/m^3^ due to its reduced thickness. Specifically, the board samples 19–21 were obtained at 7 mm thickness instead of the standard 9 mm used for all other boards, which significantly influenced the bending values even at lower density. Increasing the binder content from 21% to 25% in LS-based boards resulted in a significant improvement in both MOE and MOR values (samples 6 and 12; Table 3). However, for RE-based boards, increasing the binder content from 15% to 21% resulted in a significant decrease in bending performance (samples 25 vs. 26; Table 3), indicating that the optimal SA binder content for mechanical enhancement lies in the range of 15–20%.

#### 3.1.3. Evaluation of Adhesion

The IB values of the obtained particleboards varied substantially, ranging from 0.2 N/mm^2^ to 1.5 N/mm^2^ (Table 3), with density contributing to this variation, as confirmed by a moderate correlation (r = 0.46). No significant difference was observed between the IB values of boards made from LS furnish when increasing the SA binder from 21% to 25% or the pressing rate from 1.5 to 1.7 min/mm (samples 11 vs. 12). Although the difference between samples 12 and 13 appeared to be large (Table 3), ANOVA confirmed that it was not statistically significant due to high specimen-to-specimen variability. This further suggests that the pressing temperature, rather than pressing rate, is the dominant factor influencing IB strength.

Boards produced from RE particles under identical conditions and density achieved significantly higher IB values compared to LS particles (samples 14 vs. 26; Table 3) indicating the influence of used furnish. However, the highest IB values overall were obtained for boards made from LS-/ furnish at densities ≥ 750 kg/m^3^ (samples 9, 10). Regarding particle effects, LS-/ boards consistently outperformed LS-| boards (samples 6 vs. 14; Table 3), even when fine particles were removed (samples 8 vs. 16 and 9 vs. 17; Table 3). This can be attributed to differences in particle-size distribution between the furnishes (Table 1), in which the LS-/ furnish contains a higher proportion of fine particles (<2 mm) than LS-|, which likely improves core–layer consolidation during IB testing.

Similarly, the >2 mm fraction of RE furnish also constitutes 33%, as in LS-|, which resulted in a significantly higher IB value achieved between the boards with a density of 720 kg/m^3^ (samples 15 and 16 vs. 27), indicating that both particle shape and size distribution play important roles. The exceptionally high IB value of sample 10 (LS-/ furnish) was traced to differences in furnish preparation: a higher feed rate during log sawing reduced the proportion of fine particles (<2 mm), which—by chance—improved IB performance.

WSR was evaluated primarily for boards made under identical processing conditions but differing in density and furnish types. Despite this, WSR values varied significantly, from 209 N to 588 N (Table 3), driven mainly by density, although particle geometry also played a role. In general, boards made from RE furnish achieved higher WSR values, likely due to the thicker and harder nature of RE particles. For example, the highest WSR value (588 N) was obtained for the RE board at 720 kg/m^3^ (sample 27; Table 3). In contrast, LS boards at the same density (samples 7 and 15; Table 3) showed significantly lower WSR values, indicating the significant difference between LS and RE particles.

**Figure 5:**
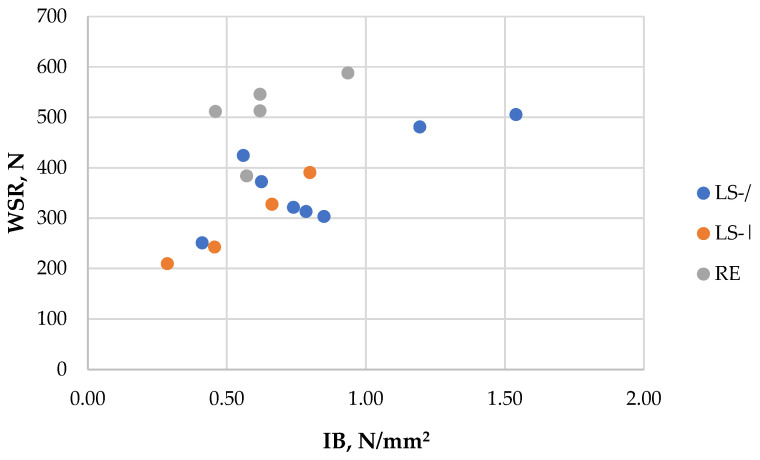
WSR vs. IB of the obtained particleboards, depending on furnish.

An exception was sample 10 (Table 3), whose altered particle-size distribution resulted in a significantly increased WSR, mirroring its IB behavior. Consistent with IB results, LS-/ boards achieved higher WSR values than LS-| boards (samples 6 vs. 14 and 7 vs. 15; Table 3). A moderate positive correlation (r = 0.47) was observed between the WSR and IB values of the obtained boards (Figure 5), indicating that the SA-based binder provides sufficiently strong internal adhesion to support WSR performance.

Regarding binder content, WSR increased slightly for LS furnish boards (samples 6 vs. 12; Table 3) but decreased for RE furnish boards (samples 24 vs. 26; Table 3). Independent of IB, WSR showed strong correlations with MOE (r = 0.82) and MOR (r = 0.76), reflecting the typical interdependence of the mechanical properties of the particleboards.

#### 3.1.4. Surface Optical Microscopy

**Figure 6:**
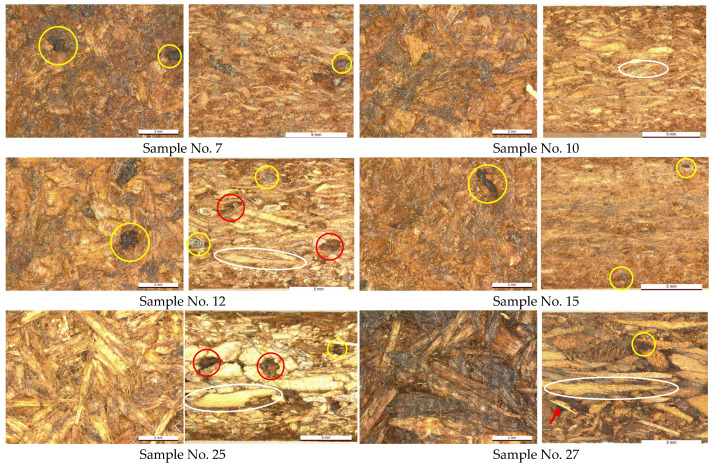
Optical surface micrographs of particleboards composed of LS (samples 7, 10, 12, 15) and RE (samples 25, 27) furnishes representing face (**left**, scale bar 2 mm) and cross sections (**right**, scale bar 5 mm).

Surface optical micrographs of the investigated particleboards are presented in Figure 6, demonstrating face and cross sections. As can be seen from Figure 6, both surfaces of the particleboards approve homogenic quality with distinct morphology of used wood particles without observed technological cracks. The face surface of the boards made from LS-/ sawdust (samples 7, 10, 12) demonstrate the view with common wood particles coated by SA binder. The small dark regions indicated by little yellow circles show that insoluble binder particles of birch outer bark remained after depolymerization and were composed of cellulose, lignin, and carbohydrates [17]. The SA binder’s solid particles can be observed on both surfaces of the particleboards. On the other hand, the bigger dark brown areas could be related to a higher level of binder penetration in some individual wood particles, indicated by the larger yellow circles in Figure 6.

The boards made from LS-| sawdust (sample 15) demonstrate more homogenous surfaces composed of particles with more even fractional distribution, complementing the results shown in Table 1 and including the higher proportion of fine particles. The homogeneity effect could also be attributed to the density of the boards, which is seen when comparing the cross-section surfaces of samples 7, 10, 15, and 27 with a density ranging from 715 to 750 kg/m^3^ vs. samples 12 and 25, which have a lower density (620–660 kg/m^3^), therefore demonstrating obvious voids between the particles (indicated by red circles in Figure 6). The significant differences in particle dimensions between LS sawdust could be observed on the cross-section surfaces comparing samples 7, 10, and 12 vs. 15. The cross-section surface of the boards also reveals the SA binder penetration into individual particles, as indicated by white oval shapes in Figure 6. The shape difference between LS and RE particles can be observed comparing particleboard samples 7, 10, 12, and 15 vs. 25 and 27 (Figure 6). The presence of laminated particles from recycled particleboard can be observed on the cross-section surface of sample 27, indicated by a red arrow. The impact of increased binder content is not observed between the boards composed of LS furnish (samples 7, 10, and 15 vs. 12), while the boards composed of RE furnish demonstrate an obvious difference with a darker color when the binder content is 25% (sample 27; Figure 6).

## 4. Discussion

In the previous section, it was shown that the properties of the obtained particleboards varied significantly depending on all the investigated factors: production conditions (pressing temperature and time), board density, and the type of furnish used. Such variability is typical for conventional particleboard as well [31]. The influence of these factors on the tested properties differed in magnitude. Board density affected all measured properties except TS, which was primarily influenced by pressing temperature and binder content. The effects of pressing conditions also varied: for example, increasing the pressing temperature from 200 °C to 220 °C slightly improved WA and IB and significantly improved TS, whereas bending properties, particularly MOE, showed a slightly negative response. The influence of all research factors on board properties, as well as the interrelationships between properties based on the calculated correlation coefficients, is summarized in Table 4. An increase in binder content showed a negative correlation with almost all board properties except IB, indicating that a higher binder content improved IB, WA, and TS. Pressing rate showed the weakest overall effect on the board properties, with a moderately negative correlation observed only for MOE (Table 4).

Overall, our conclusions are consistent with previous studies on particleboard production, including boards made from flax shives and sunflower bark bonded by casein-based adhesives [21], sawmill residues bonded with formaldehyde-based MUF adhesive [22], miscanthus bonded with synthetic MDI [32], and recycled particleboards bonded with tannin and sucrose systems [33]. Studies on furnish structure have shown that the most advantageous particleboard properties are achieved when the outer layers are formed from particles predominantly >0.63 mm and the core layer from particles >10 mm [22]. For bio-based adhesives, an optimal tannin–sucrose resin content (25:75) of 30% to 40% has been reported, along with optimal hot-pressing conditions of 220 °C and 1.1 min/mm [33].

In our study, the suitable SA binder content and pressing rate ensuring efficient board properties across all used furnishes were 21% and 1.5 min/mm, respectively. Although synthetic adhesives such as MDI ensure very fast particleboard pressing rates (approximately 25 s/mm), the MOR of the resulting boards with a density of 600 kg/m^3^ typically ranges from 7 N/mm^2^ to 10 N/mm^2^ depending on particle size distribution and composition [22]. In our case, the maximum MOR values also reached 10 N/mm^2^, but at higher densities—960 kg/m^3^ for LS furnish (sample 5; Table 3) and 750 kg/m^3^ for RE furnish (sample 21; Table 3), the latter was influenced by reduced board thickness. This suggests that the RE furnish is more compatible with the SA binder; however, the LS furnish also shows strong potential, particularly in terms of water resistance and adhesive performance. The advantage of RE furnish could be characterized by the larger particle dimensions, especially in the core layer of the board, as was shown in Figure 6.

The favorable water resistance and bonding performance of SA-bonded particleboards can be attributed to the efficient depolymerization of birch outer bark, yielding substantial amounts of monomeric SA, monosaccharides, and polyphenolic compounds [17]. These components cross-link effectively with lignocellulosic particles under appropriate hot-pressing conditions, resulting in good physical–mechanical properties. The insoluble SA binder solid particles composed of lignocellulosic complex also showed good integrity without detected technological cracks inside the particleboard, as presented in Figure 6 by micrographs. The WSR values of the most advanced particleboards obtained in this study (samples 10 and 27; Table 3) achieved values between 505 and 588 N, comparable to the WSR of soybean-based boards bonded with epoxidized sucrose soyate, which vary between 68 N and 680 N [34]. Previous work on particleboards made from SA residues and different pine particles has shown that WSR is strongly dependent on particle size, with variations of up to 50%, and the highest values were obtained with coarse particles bonded with 20% SA content [18].

Regarding the wood species used in this study, the achieved particleboard properties are comparable to those obtained from birch wood particles bonded with the same SA binder [15]. The penetration of the SA binder into the pine particles was obviously observed by microscopy, as shown in Figure 6. Only the bending properties were lower than those reported for birch wood-based boards (MOR up to 18.7 N/mm^2^); however, those results were affected by higher board density (>800 kg/m^3^) despite variations in binder parameters [17]. Particleboards produced from SA residue (20%) and birch particles at a density of ~850 kg/m^3^ achieved a maximum MOR of 11.4 N/mm^2^ and MOE of 3518 N/mm^2^, which was attributed to the high pressing temperature (250 °C) combined with a relatively low pressing rate (0.7 min/mm) [16]. In contrast, particleboards made from the same SA residue and pine wood particles at a density of 700 kg/m^3^ demonstrated MOR and MOE values ranging from 3.7 N/mm^2^ to 6.4 N/mm^2^ and 164 N/mm^2^ to 2205 N/mm^2^, respectively, depending on binder content (10–20%) and particle type (fine, medium, coarse) [18].

In that study, the maximum MOE was achieved using coarse particles—similar to those used in industrial-oriented strand board production, which are not usually used in conventional particleboard production. However, the MOR of boards made from coarse pine particles was only 4.2–4.5 N/mm^2^, significantly lower than that of boards made from medium particles (5.8–6.4 N/mm^2^). These values closely align with our results, indicating the strong effect of particle geometry and the inherent limitations of pine particles resulting in a relatively lower bending performance. Comparing the available studies, it is likely that the achieved poor bending properties in our work were affected by insufficient pressing temperature and an overly long pressing rate. As noted earlier, particleboards made from birch wood particles bonded with SA binder achieved their highest bending properties at temperatures between 230 °C and 250 °C and a pressing rate of 0.7 min/mm, although these results were also associated with higher board densities [15,16]. From another perspective, the CWM blocks made of sawn pine solid wood—the source of the LS sawdust used in this study—exhibit MOR and MOE values of only 2.15 N/mm^2^ and 140 N/mm^2^, respectively [23], which are substantially lower than the values achieved in our particleboards.

With respect to EN requirements for particleboards intended for interior fitments, including furniture [24], not all properties obtained in this study met the standard requirements. Actually, only the bending properties fell slightly below the required values. Notably, the board composed of RE particles at a density of 750 kg/m^3^ demonstrated the most suitable manufacturing parameters, achieving the required value for MOE (sample 21; Table 3). This achievement indicates promising potential for the future development of SA-bonded particleboards, particularly through adjustments in furnish configuration to enhance bending performance. It is worth noting that particleboards produced from agricultural biomass combined with various adhesive systems—including formaldehyde-based resins—often exhibit MOR values below the EN standard requirement (11 N/mm^2^) [20].

## 5. Conclusions

This study demonstrates that particleboards can be successfully produced from both LS and RE furnishes using a SA-based natural binder derived from birch outer bark. The SA binder shows clear potential for manufacturing particleboards from pine sawdust—both virgin and recycled—supporting a more sustainable use of wood resources. Among the investigated factors, furnish composition, thickness, density, and pressing temperature had the strongest influence on the resulting board properties.

Particleboards manufactured from RE furnish with 21% SA binder content and hot-pressed at 220 °C for 1.5 min/mm—with a final thickness of 7 mm and a density of 750 kg/m^3^—approached the performance requirements specified in EN 312 for Type P2 interior fitments. The optimal properties achieved in this study (TS 9–12%, MOE 1700–2040 N/mm^2^, MOE 7.9–10.1 N/mm^2^, IB 0.72–1.54 N/mm^2^) indicate that SA-bonded boards have promising potential, particularly when furnish configuration and pressing parameters are further optimized. The performed optical microscopy approved the good homogeneity of particleboard components, including SA penetration in particles without observed technological cracks.

Future work should focus on improving bending performance, as well as evaluating long-term durability and cost efficiency to support the broader industrial adoption of SA-bonded particleboards.

## Figures and Tables

**Table 1:** Percentage of used furnish particle dimensional fractions.

Furnish	<0.5 mm	0.5–1 mm	1–2 mm	>2 mm
LS-/	19.4	20.7	43.5	16.2
LS-|	35.3	17.6	14.6	32.4
RE	12.8	27.6	26.2	33.4

**Table 2:** Parameters of particleboard production.

Furnish	Binder (%)	Temperature (°C)	Pressing Rate (min/mm)	Density (kg/m^3^)
LS	21–25	190–220	1.0–1.7	650–850
RE	15–21		0.9–1.5	650–750

**Table 4:** Correlation coefficients (r) between the research factors and particleboard properties.

Property	Binder	Density	Temperature	Pressing Rate	WA	TS	MOE	MOR	IB
WA	−0.33	−0.73	−0.39	0.19	1	0.52	−0.38	−0.70	−0.71
TS	−0.74	−0.02	−0.67	−0.12	0.52	1	0.50	0.04	−0.33
MOE	−0.60	0.51	−0.15	−0.40	−0.38	0.50	1	0.79	0.28
MOR	−0.15	0.65	0.06	−0.13	−0.70	0.04	0.79	1	0.66
IB	0.28	0.46	0.28	0.16	−0.71	−0.33	0.28	0.66	1
WSR	−0.48	0.24	−0.21	0.02	−0.25	0.52	0.82	0.76	0.47

## Data Availability

The original contributions presented in this study are included in the article. Further inquiries can be directed to the corresponding author.

## Abbreviations

The following abbreviations are used in this manuscript:

CWM	Cellular wood material
EN	European standard
IB	Internal bonding
LS	Sawdust obtained by longitudinal sawing
MC	Moisture content
MOE	Modulus of elasticity
MOR	Modulus of Rupture
RE	Recycled particles
SA	Suberinic acids
TS	Thickness swelling
WA	Water absorption
WSR	Withdrawal screw resistance
